# Phosphates form spectroscopically dark state assemblies in common aqueous solutions

**DOI:** 10.1073/pnas.2206765120

**Published:** 2022-12-29

**Authors:** Joshua S. Straub, Mesopotamia S. Nowotarski, Jiaqi Lu, Tanvi Sheth, Sally Jiao, Matthew P. A. Fisher, M. Scott Shell, Matthew E. Helgeson, Alexej Jerschow, Songi Han

**Affiliations:** ^a^Department of Physics, University of California, Santa Barbara, CA 93106-9530; ^b^Department of Chemistry, University of California, Santa Barbara, CA 93106-9510; ^c^Department of Chemistry, New York University, New York, NY 10003; ^d^Department of Chemical Engineering, University of California, Santa Barbara, CA 93106-5080

**Keywords:** phosphate, assembly, dark state, dehydration

## Abstract

Phosphates and polyphosphates play ubiquitous roles in biology as integral structural components of cell membranes and bone, or as vehicles of energy storage via adenosine triphosphate and phosphocreatine. The solution phase space of phosphate species appears more complex than previously known. We present nuclear magnetic resonance (NMR) and cryogenic transmission electron microscopy (cryo-TEM) experiments that suggest phosphate species including orthophosphates, pyrophosphates, and adenosine phosphates associate into dynamic assemblies in dilute solutions that are spectroscopically “dark.” Cryo-TEM provides visual evidence of the formation of spherical assemblies tens of nanometers in size, while NMR indicates that a majority population of phosphates remain as unassociated ions in exchange with spectroscopically invisible assemblies. The formation of these assemblies is reversibly and entropically driven by the partial dehydration of phosphate groups, as verified by diffusion-ordered spectroscopy (DOSY), indicating a thermodynamic state of assembly held together by multivalent interactions between the phosphates. Molecular dynamics simulations further corroborate that orthophosphates readily cluster in aqueous solutions. This study presents the surprising discovery that phosphate-containing molecules, ubiquitously present in the biological milieu, can readily form dynamic assemblies under a wide range of commonly used solution conditions, highlighting a hitherto unreported property of phosphate’s native state in biological solutions.

## Significance

This study presents an unexpected discovery that phosphate-containing molecules form dynamic assemblies under common biologically relevant aqueous solution conditions. Phosphates are ubiquitous in the biological milieu in the form of free phosphate ions, phosphorylated proteins, RNA, DNA, ATP, the cell membrane, and calcium phosphate species en route to bone formation. The discovery that phosphate- containing species, including orthophosphates, can readily assemble in water is important for understanding the many complex processes in which they are involved and should be considered when studying their role in modulating biocatalysis, cellular energy balance, or the formation of biomaterials.

Phosphate-containing species are in constant flux throughout the phosphorus cycle and accumulate within the cells of all living organisms. Cellular energy is primarily harvested through the dynamical formation and breakage of phosphoanhydride chemical bonds of adenosine phosphates (1, 2). Free phosphates and their subsequent assembly are also involved in bone formation and growth (3–5); however, the underlying assembly mechanisms of phosphate species and other ions that lead to bone formation processes are not well understood. An understanding of the equilibrium between free phosphates and higher-order phosphate assemblies in the form of polyphosphates and phosphate clusters would provide further insight into the mechanisms involving biological energy storage and/or the engineering of biological structures.

^31^P nuclear magnetic resonance (NMR) offers useful information about the composition, dynamics, and structural properties of lipid membrane interfaces (6–8), phosphorylated biomolecules (9–11), polyphosphates (12, 13), and precursors of bone formation (14). We performed ^31^P NMR to investigate the native state of phosphate species as a function of temperature with the initial intent to subsequently study the formation processes of calcium phosphate clusters. In this process, we encountered peculiar ^31^P NMR line broadening with increasing temperature of aqueous solution of pure phosphates. Such characteristics cannot be explained by the usual temperature-dependent *T*_2_ relaxation due to increasing molecular tumbling of small molecules with increasing temperature. ^31^P NMR line broadening as a function of pH, phosphate concentration, and counter-cation species has been described in the literature (15–17); however, line broadening with increasing temperature has not been reported before.

Underscoring these unexpected observations, we present experimental results showing that phosphate-containing species, including orthophosphate, pyrophosphate, and adenosine diphosphate assemble into hitherto unreported spectroscopically “dark” species, whose fractional population increases with increasing temperature. This observation is shown to be consistent with the dehydration entropy-driven formation of dynamic phosphate assemblies. ^31^P NMR chemical exchange saturation transfer (CEST) reveals that phosphates assemble into species with broad spectroscopic signatures, whose

population is in exchange with NMR-detectable phosphate species. A subpopulation of these assemblies is also observed in cryogenic transmission electron microscopy (cryo-TEM) images to exhibit droplet-like spherical assemblies up to 50 nm in diameter. The discovery that common phosphate-containing molecules can readily assemble into higher-order species in water under physiological conditions in the absence of biologically activated processes should be relevant to a variety of biological and biochemical processes that use phosphate-containing species as building blocks, energy sources, or reactants in an aqueous environment.

## Results and Discussion

### Unexpected NMR Relaxation Behavior.

**Fig. 1.**
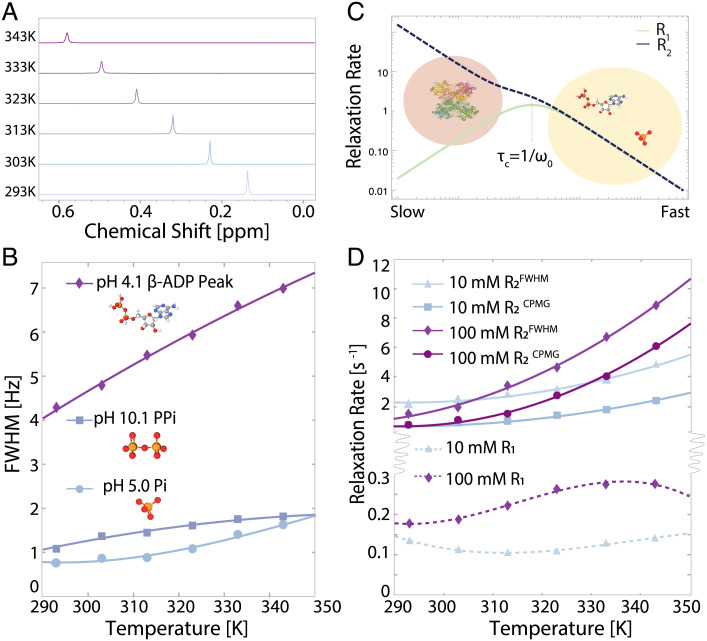
^31^P NMR results for phosphate-containing species. (*A*) 1D NMR spectra from 10 mM sample in (*D*) taken at every 10 K showing line broadening in orthophosphate. (*B*) Linewidths for orthophosphate, pyrophosphate, ADP, and ATP as a function of temperature showing monotonic increase with temperature. Solid lines are quadratic fits to data to guide the eye. (*C*) *R*_1_ and *R*_2_ curves as a function of molecular tumbling rate from Bloembergen–Purcell–Pound theory. Cartoons illustrate the approximate locations of ionic phosphate, ADP, and a standard protein based on tumbling rates. (*D*) *R*_2_ as extracted from a CPMG pulse sequence and from FWHM for 10 mM and 100 mM monobasic sodium orthophosphate pH 4.5 as a function of temperature, showing monotonic increase in *R*_2_ in each case. Solid lines are quadratic fits to data to guide the eye. *R*_1_ for 10 mM, 100 mM, monobasic sodium orthophosphate pH 4.5 as a function of temperature showing different curve shapes as a function of concentration. Solid lines are cubic fits to data to guide the eye.

A series of ^31^P NMR spectra were acquired of an aqueous solution of sodium orthophosphate at 10 mM concentration, pH 4.5 and as a function of temperature between 293 K and 343 K. Each spectrum consists of a single ^31^P NMR line that shows significant broadening with increasing temperature, as shown in Fig. 1*A*. The full width at half maximum (FWHM) linewidth increases from 0.74 to 1.21 Hz, while the chemical shift only slightly changes from 0.14 to 0.58 ppm as referenced to 85% H_3_PO_4_ at 293 K. To test the consistency and generality of this observation, these measurements were repeated for solutions of orthophosphates at concentrations of 100 mM and 1 M, at varying pH from 4 to 10, at field strengths corresponding to ^1^H NMR frequencies of 400 MHz and 500 MHz, and for solutions of orthophosphates with sodium and potassium counterions, as shown in *SI Appendix*, Figs. S1–S4. Under every condition tested, the general trend of ^31^P NMR line broadening with increasing temperature was observed.

To further explore this observation, we tested a series of phosphate-containing species in addition to orthophosphates, such as pyrophosphate, adenosine diphosphate (ADP), and adenosine triphosphate (ATP). While the extent of ^31^P NMR line broadening with increasing temperature varies between the different species and solution conditions, the general trend of line broadening with increasing temperature persists for all phosphate-containing species tested here (Fig. 1*B*), suggesting that there is a common underlying molecular mechanism for solvent-exposed phosphate groups.

This line broadening is surprising, as it is inconsistent with expected trends for small molecules, including ionic species. Increasing temperature should generally lead to motional narrowing of NMR resonances of small molecules as their tumbling rate increases. An exception to this trend would be a case where increasing chemical exchange rate leads to a transition from an intermediate to a faster exchange regime, where the chemical shifts of the two species significantly diverge, given that the line width is proportional to the square of the chemical shift difference. In such a case, however, one would normally observe the splitting of the broad line into additional narrow resonances at sufficiently high temperatures, which was not observed for any of the phosphate-containing species studied under a wide range of experimental conditions. An additional possibility is scalar relaxation of the first kind, which has been observed to lead to line broadening with increasing temperature (18). However, for such a case, the proton-exchange rate should be of the same order of magnitude as the linewidth, i.e., on the order of a few Hz, which is not the case for our phosphate solutions, given that the second-order rate constant for proton exchange between monobasic and dibasic phosphate is 1.45  *  10^9^ mole^-1^ l s ^-1^, larger than Hz (19).

To further examine the nature of the underlying process leading to the observed line broadening and its temperature-dependence, we measured the ^31^P NMR spin-spin relaxation rate, *R*_2_, at varying temperatures from 293 K to 343 K. This approach allowed us to assess whether the ^31^P NMR line broadening with increasing temperature originated from inhomogeneous broadening due to the presence of multiple distinct spectral components or lifetime broadening. The value for *R*_2_ in Hz measured by the Carr–Purcell–Meibom–Gill (CPMG) (20, 21) sequence was compared to that extracted from the FWHM (following *R*_2_ = *π*⋅ FWHM) for a 10 mM and 100 mM solution of sodium orthophosphate, as shown in Fig. 1*D*. We found that the two showed comparable trends for broadening, with slightly higher (1 to 2 Hz) values for the FWHM-derived linewidth compared to that directly measured via *R*_2_, likely due to field inhomogeneities and small temperature gradients. This observation verified that the phosphate linewidth is primarily broadened by the dynamical properties of a homogeneous spectral population. This correspondence was found consistently across all samples tested. The expected trend from Bloembergen–Purcell–Pound (BPP) theory (22) of decreasing *R*_2_ with increasing molecular tumbling rate, i.e., temperature, is shown in Fig. 1*C* to contrast to the experimental trend shown in Fig. 1*D*.

The temperature-dependence of the spin-lattice relaxation rate, *R*_1_, provides further information on the molecular-scale dynamical properties of these phosphate solutions. We measured *R*_1_ for a series of phosphate concentrations and again observed unexpected values and trends. As illustrated in Fig. 1*C*, small molecular species tumble in the “fast” regime—where rotational correlation time is faster than the Larmor frequency—and so *R*_1_ is expected to monotonically decrease with increasing temperature and to be nearly identical with the *R*_2_ values. Instead, we observe *R*_1_ values for orthophosphates at concentrations from 10 mM to 100 mM that are as many as one to two orders of magnitude smaller than the *R*_2_ values of the same samples. This observation suggests that the monitored phosphate species experience much slower dynamics than those of isolated orthophosphate monomers. Assuming a random field relaxation mechanism, the molecular tumbling correlation time would have to be larger than 10 ns, corresponding to a hydrodynamic diameter larger than 4.4 nm according to the Stokes–Einstein relation in order to result in the observed difference between *R*_1_ and *R*_2_.

This consideration leads to the question of whether the states of phosphates giving rise to the observed properties correspond to phosphate assemblies. When examining the shape of change in *R*_1_ with increasing temperature, we observed a subtle deviation from BPP theory for the solution of sodium orthophosphates at 10 mM concentration (Fig. 1*D*). The initial decrease of *R*_1_ with temperature is expected but not the observed increase at temperatures above 310 K. This latter observation is again consistent with a temperature-induced formation of larger phosphate assemblies, as such assemblies would lead to a lower rotational correlation time, effectively moving in the direction of the “slower” motion regime (toward the left of the x axis) as illustrated in Fig. 1*C*. A similar trend is observed for 10 mM potassium orthophosphate samples (*SI Appendix*, Fig. S1).

The temperature dependence of *R*_1_ for sodium orthophosphates at higher concentrations (100 mM) showed a local maximum with increasing temperature (Fig. 1*D*).This trend is again inconsistent with the dynamical properties of small molecules in solution. According to the BPP theory, a local maximum in *R*_1_ is expected only for species with rotational correlation times, *τ*_*c*_ matching the inverse nuclear Larmor frequency, *ω*_0_ (Fig. 1*C*). At 11.7 T and a ^31^P NMR frequency of $\frac{\omega_{0}}{2\pi }$ = 200 MHz, assuming a random field relaxation mechanism, we estimate *τ*_*c*_ ∼ 800 ps following *τ*_*c*_ = $\frac{1}{\omega_{0}}$. A rotational correlation time in this range implies a particle diameter of 2 nm for a spherical object according to the Stokes–Einstein relation. Regardless of the exact shape of the species, this size is several fold larger than that of monomeric orthophosphates (23).

The observed temperature-dependent trends in *R*_1_ and *R*_2_ are consistent with (*A*) the phosphate molecules assembling into larger species, whose tumbling rate lies in the slow motion regime, with correlation time *τ*_*c*_ exceeding *ω*_0_, or (*B*) phosphate molecules being in exchange with spectroscopically invisible species that have much higher *R*_1_ and *R*_2_ rates. Explanation (*B*) would again be consistent with phosphate assemblies since there are no other constituents than phosphate ions in the solution. Higher temperatures may facilitate the growth in population and size of such assemblies and/or accelerate the exchange and hence enhance *R*_1_ and *R*_2_ of the detected ^31^P NMR signal. It is also possible that monomeric phosphates coexist with spectroscopically invisible phosphate clusters across the temperature range tested and that heating increases the relative abundance of this invisible species. Either scenario suggests the formation of larger phosphate assemblies, with enhanced populations and/or exchange rates at elevated temperatures, yielding much greater *R*_2_ values compared to *R*_1_ and consistent with our observed trends in relaxation with temperature. Notably, after cooling the sample that was heated back down to its initial temperature, the relaxation rates return to their initial values (*SI Appendix*, Fig. S5), suggesting that the assembly formation is reversible.

If larger assemblies are forming, it is important to consider their nature and, in particular, the interactions leading to their formation. One possibility is that the new assemblies are polyphosphates formed by the enhanced formation of P–O–P bonds at elevated temperatures. The ^31^P chemical shift for phosphates is known to shift by approximately −10 ppm with each P–O–P bond formed and by a maximum of 5 ppm from the unprotonated to triply protonated states (24, 25). This is inconsistent with our observed chemical shifts that move systematically downfield, but only very slightly, by a maximum of 0.5 ppm when the temperature is increased from 293 K to 343 K. Hence, the observed chemical shift change is too small to be attributed to covalent P–O–P bond formation. The observed 0.5 ppm chemical shift change could instead be the result of changes in the equilibrium P–O bond length, potentially induced by the noncovalent association of phosphate molecules. Such changes could be mediated by hydrogen bond interactions that, in turn, can be modulated by changes in phosphate hydration. Notably, all four oxygens of the phosphate group can serve as hydrogen bond donors or acceptors, depending on the protonation, hydration, and partial charge state of the group, hence allowing for multivalent interactions that can give rise to the formation of larger assemblies, while still maintaining rapid exchange with ionic phosphates and small clusters held together by weak interactions. In any case, the species forming must either have the same chemical shift as the orthophosphate ions and/or be so broad as to be rendered spectroscopically invisible.

### Indirect Observation of Phosphate Assemblies by ^31^P CEST.

To test whether the phosphate species are in exchange with a spectroscopically dark population, we performed CEST experiments. CEST typically provides a means of identifying signatures of exchangeable species with distinct chemical shifts from the visible species but below the direct NMR detection limit. This effect is achieved by saturating a selected region in the spectroscopically invisible region of the spectrum, followed by the detection of the (visible) signal of a major species (in this case, monomeric phosphates) that is in exchange with the species below the NMR detection limit. Repeating these experiments with different saturation frequencies across the complete spectral region and saturation power permits the scanning of an entire spectrum for potentially exchanging species. This procedure has been widely employed, for example, to identify weakly populated states of peptides and proteins whose protons are in exchange with water (26, 27) and in this context is often referred to as DEST (for dark state exchange saturation transfer) (28). The sensitivity enhancement effect for the dark species is achieved because exchange can occur many times during the saturation pulse and thereby transfer saturation levels between the visible and invisible species repeatedly. In the CEST experiment of this study, we recorded ^31^P NMR spectra of the visible ^31^P NMR signals following rf irradiation (with nutation frequency of 150 Hz for 5 s) at a specified resonance frequency in what can be seen as a one-dimensional pump–probe experiment. Here, the pump frequency is stepped through a frequency range of approximately 8,000 Hz, centered around the one visible ^31^P NMR peak. In this fashion, CEST can test for the existence of spectroscopic dark states that are in exchange with phosphate species at frequencies within the scanned range. A plot of the detected intensity vs. saturation frequency offset is called a Z-spectrum.

**Fig. 2.**
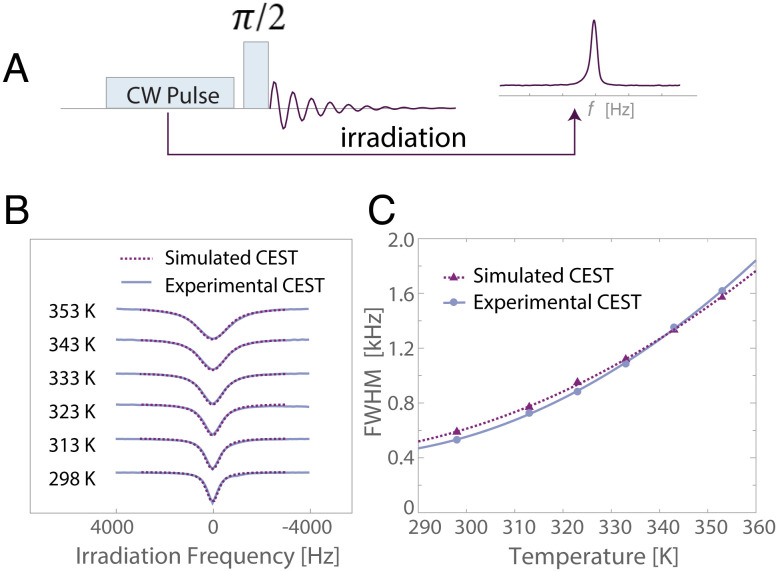
^31^P CEST results for 100 mM orthophosphate (pH = 4.5). (*A*) CEST pulse sequence. (*B*) The experimental and simulated CEST Z-spectra as a function of temperature with 150 Hz of the irradiation pulse power. (*C*) The width at half height of both the experimental and simulated Z-spectrum dips from (*B*) as a function of temperature. The solid and dashed lines represent a quadratic fit to data as a guide for the eye.

Fig. 2 *B* and *C* shows both the measured (solid lines) and the simulated (dashed lines) Z-spectra as a function of temperature of a 100 mM orthophosphate solution. It is seen clearly that the widths of the dips in the Z-spectra increase with increasing temperature, which is consistent with the unexpected trend observed with the *R*_2_ and linewidth data.

In the absence of exchange, one expects the width of the dip in the Z-spectrum (at half maximum) to be approximately a factor two times the rf nutation frequency (here 150 Hz) (29). In all cases for CEST measurements (both as a function of temperature and as a function of irradiation power), we observed the dip widths to be significantly larger (a factor of 3 to 10) than the expected twofold the nutation frequency (*SI Appendix*, Fig. S10). This finding is a clear sign that there must be significant underlying exchange processes with spectroscopically silent species and that these processes change with temperature.

These results further corroborate the assumption that exchange occurs with a population exhibiting a broad spectroscopic signature, which is invisible to direct spectroscopic detection. The relative population of this spectroscopically “dark” species may also be increasing with increased temperature. When the solutions are cooled back down to 298 K after heating, the Z-spectrum dip width returns to the originally measured value (*SI Appendix*, Fig. S6), indicating reversible assembly formation. Similar CEST results were observed for a range of pH values of orthophosphate solutions (*SI Appendix*, Fig. S7) and for ADP (*SI Appendix*, Fig. S8), suggesting that this behavior is common among several phosphate-containing molecular species in aqueous solutions.

In order to further substantiate the hypothesis and generate a potential model, simulations were performed that could simultaneously satisfy the values and temperature trends of *R*_2_ and CEST data. For the simulation, a two-pool model was used, with *A* referring to the detectable pool (phosphate monomers) and *B* referring to the spectroscopically silent pool (the assemblies). The model used separate *R*_1_ and *R*_2_ for pools *A* and *B*, exchange rate constants for the forward and backward interconversion processes *A* ↔ *B*, and the relative populations of the pools, considering first-order kinetics. The rate constants were assumed to be of Arrhenius type, and the forward rate constant was parameterized using an Arrhenius prefactor *k*_0_ and activation energy *E*_*a*_. The change of enthalpy *ΔH*_*PA*_ and change of entropy *ΔS*_*PA*_ for assembly formation derived from this simulation were then used to find the assembly population, *p*_*B*_, using the Boltzmann factor *p*_*B*_ = exp(−*ΔG*_*PA*_/(*RT*))/(1 + exp(−*ΔG*_*PA*_/(*RT*))), where *ΔG*_*PA*_ = *ΔH*_*PA*_ − *TΔS*_*PA*_. The temperature dependence entered through the *TΔS*_*PA*_ term, while *ΔH*_*PA*_ and *ΔS*_*PA*_ were assumed constant. At each temperature, these relationships were used to calculate the forward and backward exchange rates, and the McConnell equations were solved iteratively to satisfy the experimental *R*_2_ and CEST data using the Spinach software package (30).

Simulations of the *R*_2_ data as a function of temperature of the 100 mM NaH_2_PO_4_ sample (at pH = 4) led to the following parameters to describe the exchange process: *ΔH*_*PA*_ = 25 kJ/mol, *ΔS*_*PA*_ = 30 J/(mol K), *k*_0_ = 20, 000 s^−1^, and *E*_*a*_ = 10 kJ/mol. We then calculated the relative fraction for the B population, *p*_*B*_, as described earlier. The forward and backward exchange rates are obtained via *k*_*f*_ = *k*_0_exp(*E*_*a*_/*RT*) and *k*_*b*_ = *k*_*f*_(1 − *p*_*B*_)/*p*_*B*_, in steady state. For CEST simulations, *R*_1_ was set equal to the measured values (shown in Fig. 1) for both pools (after verifying that setting *R*_1_^*B*^ = 0.1*R*_1_^*A*^ to 10*R*_1_^*A*^ did not change the quality and results of the fit), and *R*_2_^*A*^ was set equal to *R*_1_^*A*^, assuming a fast motion regime for the small molecular entity. Modeling of the *R*_2_ data indicated that *k*_*f*_ ≫ *p*_*B*_*ΔR*_2_ is likely, where *ΔR*_2_ = *R*_2_^*B*^ − *R*_2_^*A*^. In this regime, *R*_2_ ≈ *p*_*B*_*ΔR*_2_ (31). We therefore used this expression to determine the *R*_2_^*B*^ values for the CEST simulation by using the fitted *p*_*B*_ values and the experimental *R*_2_ values and relying on *R*_1_^*A*^ = *R*_2_^*A*^, i.e., by equating *R*_2_^*A*^ to the experimental *R*_1_ values. These values ranged from 450 to 1,000 s^−1^ over the experimental temperature range (increasing with increasing temperature), corresponding to linewidths exceeding 1,400 Hz. Such widths make the signals fall signifcantly below the detection limit.

Using this approach, a consistent model was found to fit concurrently the experimental *R*_2_ and CEST data (see Fig. 2 and *SI Appendix*, Fig. S10). The key takeaway is that increasing temperature leads to either larger or less mobile assemblies, as reflected in increasing *R*_2_^*B*^ with temperature (*SI Appendix*, Fig. S11), an increase in both *k*_*f*_ with increasing temperature (*SI Appendix*, S10*A*), with the rates spanning 350 to 660 Hz. Most critically, the model that describes the *R*_2_ and CEST data finds the fractional population of the assemblies, *p*_*B*_, to be very small but to grow with temperature (*SI Appendix*, S10*C*), with *p*_*B*_= 0.0013 at 293 K and 0.0073 at 353 K. While the absolute value of *p*_*B*_ in a dilute solution of monophosphates is small, the size of the assembly (the B pool) may comprise a very large number of monomers (the A species), as reflected in the extremely high *R*_2_^*B*^ rates. Assemblies formation, even with a small *p*_*B*_, can have a significant impact on biological assemblies between phosphate-containing species in closer proximity.

### Cryo-TEM.

**Fig. 3.**
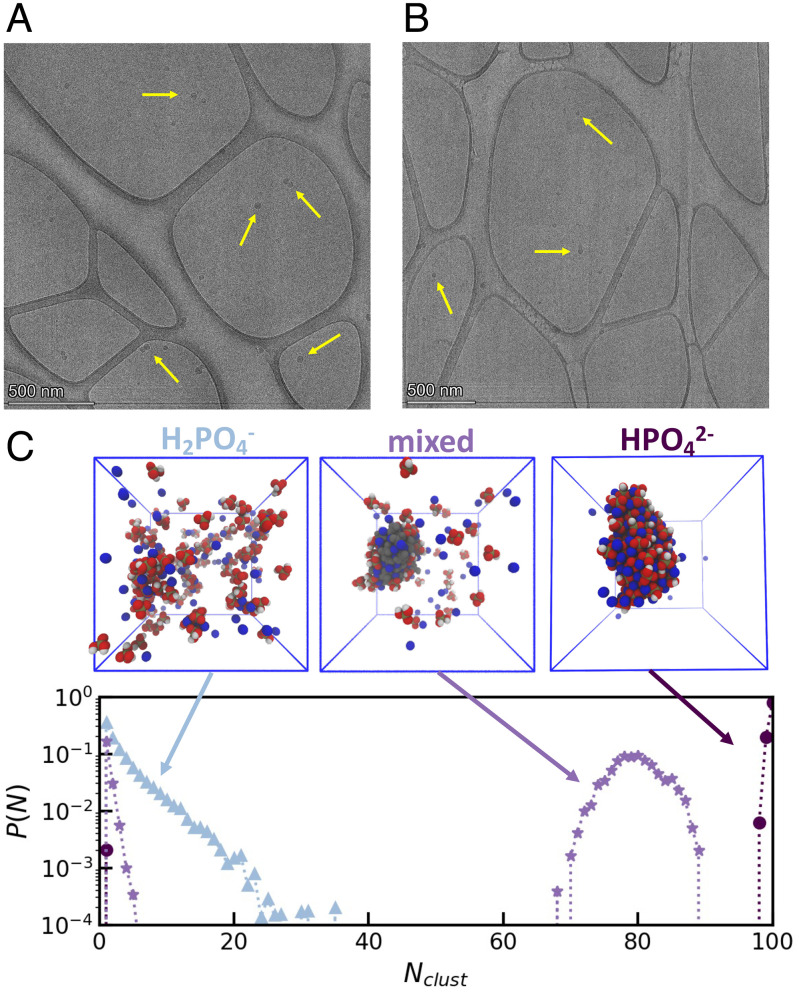
Evidence of phosphate assemblies from TEM and MD simulations. (*A* and *B*) TEM images of phosphate assemblies (yellow arrows) after heating phosphate solutions show droplet-like features forming at 25 to 50 nm in size. Samples were from different sources and prepared on different days. (*A*) 100 mM potassium ADP heated to 343 K before vitrification. (*B*) 100 mM sodium ADP heated to 343 K before vitrification. (*C*) Cluster size distributions from MD simulations at 343 K show the fraction, *P*(*N*), of phosphate ions in a cluster of size *N*_*clust*_. The insets show snapshots of phosphate assemblies (red and white) and sodium ions (blue) from the simulations. The cluster size distribution and snapshots show that HPO_4_^2−^ strongly assembles in contrast to H_2_PO_4_^−^. When H_2_PO_4_^−^ is mixed with HPO_4_^2−^, the latter induces clustering of H_2_PO_4_^−^. In this mixed system, the HPO_4_^2−^ ions are grayed out to highlight the clustering of H_2_PO_4_^−^. Simulation snapshots are visualized using Visual Molecular Dynamics (34).

While there is compelling evidence for assembly formation, the previous measurements did not provide direct observation of the assemblies due to their spectroscopically dark nature. We hence used cryogenic transmission electron microscopy (cryo-TEM) to determine whether the phosphates assemble into large and persistent enough clusters to be imaged. Cryo-TEM was performed on ADP solutions that were vitrified after heating for at least 48 h. Phosphate-containing solutions tested showed evidence of assemblies forming at diameters ranging from 30 nm to 50 nm in diameter (Fig. 3 *A* and *B* and *SI Appendix*, Fig. S12). This is in contrast to a 500 mM KCl control solution, where a majority of features were at diameters of 50 nm and greater, consistent with the consensus within previous cryo-TEM literature establishing that the most abundant form of ice artifacts are at sizes > 50 nm (32, 33). Given that these samples were prepared under identical conditions, we take this difference in the 30 to 50 nm region of the particle size distributions between the phosphate-containing solutions and the KCl control to indicate the presence of phosphate assemblies. Amongst the conditions tested, the abundance of assemblies appeared higher in solutions heated at 343 K compared to solutions that are unheated or salt controls without phosphate present (*SI Appendix*, Fig. S13). Additionally, ADP samples from different sources, and prepared on different days, showed an abundance of these features (Fig. 3 *A* and *B* and *SI Appendix*, Fig. S12). Such analysis of cryo-TEM micrographs cannot provide quantitative analysis of the whole sample, given the nature of the thin film of water that forms before vitrification that can affect which particles may be imaged. Still, this methodology provides evidence that these features are not artifacts of sample preparation. We also found that for samples of 100 mM and 1 M sodium monophosphate, the size distribution of structures is independent of phosphate concentration (*SI Appendix*, Fig. S14), providing additional evidence that these are equilibrium structures. Notably, the entire phosphate-containing population is part of or is in exchange with the assemblies, given the homogeneously broadened nature of the ^31^P NMR line. This suggests that the monomeric ADP and monophosphate populations are at thermodynamic equilibrium with the assemblies that may exist as liquid droplets, given their spherical shape. The phosphate assembly may be driven by liquid–liquid phase separation. However, further validating such a hypothesis is outside the scope of this study, given that the assemblies evade quantitative analysis.

### Molecular Dynamics Simulations.

We next used molecular dynamics (MD) simulations to probe whether monomeric phosphate species can form stable clusters under the relevant aqueous solution conditions. We simulated three different solutions of orthophosphates: HPO_4_^2−^, H_2_PO_4_^−^ and a 1:1 mixture of HPO_4_^2−^ and H_2_PO_4_^−^ using a modified GAFF forcefield (35) and TIP3P water (36). The simulated systems each contain 100 total orthophosphates, enough Na^+^ ions to neutralize them (200, 100, and 150, respectively), and water molecules to solvate to approximately 1 M orthophosphate concentration (4,759, 4,852, 4,801 water molecules, respectively). We also simulated two less concentrated systems (∼78 mM), comprising 1) 3 HPO_4_^2−^ ions, 6 Na^+^ ions, and 2,149 water molecules and 2) 3 H_2_PO_4_^−^ ions, 3 Na^+^ ions, and 2,149 water molecules to more carefully probe the temperature-dependent water–phosphate, phosphate–phosphate, and counterion–phosphate interactions. Additional details regarding the systems and simulation workflow are provided in *SI Appendix*, Table S1. At 343 K, the HPO_4_^2−^ system shows a strong tendency to assemble, forming a cluster comprising all 100 phosphate ions in the simulation box. The cluster size distribution for the HPO_4_^2−^ system, represented as the fraction, *P*(*N*), of phosphate ions in clusters of size *N*_*clust*_, is therefore peaked at the maximum number of HPO_4_^2−^ ions of 100 (Fig. 3*C*). We hypothesize that the equilibrium cluster size is larger than accessible by atomistic MD. This result is consistent with the observation of large phosphate assemblies visible to cryo-TEM, giving rise to distinct NMR spectral and relaxation properties. Interestingly, the H_2_PO_4_^−^ system shows a much weaker tendency to assemble, with a peak in the cluster size distribution at *N*_*clust*_ = 1, indicating a preference to remain unaggregated at these conditions. However, in the mixed system, the presence of HPO_4_^2−^ induces assembly of H_2_PO_4_^−^ ions, and the system forms an assembly that comprises all 50 HPO_4_^2−^ in the simulation box and some H_2_PO_4_^−^ (*SI Appendix*, Fig. S16 *B*–*D*). The three systems exhibit qualitatively the same behavior at 293 K (*SI Appendix*, Fig. S16*A*). The simulations are limited to system sizes smaller than the experimentally observed assemblies, as well as constant ionization state (as opposed to constant pH), which limits our ability to draw conclusions regarding the temperature dependence and size distributions of the observed clustering behavior. However, the MD simulations do confirm that orthophosphates cluster under comparable conditions as experimentally probed.

In addition, the sodium ions were observed to be involved in the phosphate assemblies by MD simulations, and therefore, the linewidth of sodium in the presence and absence of phosphate was investigated. While the ^23^Na NMR line was found to narrow with increasing temperature, the ^23^Na linewidth was broader for samples of NaCl with phosphate present as compared to NaCl without phosphates at both 298 K and 343 K (*SI Appendix*, Fig. S17). This observed increase in linewidth for ^23^Na when phosphate is added to solution is consistent with the sodium ions also being incorporated into the phosphate assemblies. Although ^23^Na still narrows with temperature, this can be explained by the mechanisms that traditionally lead to line narrowing at higher temperature (such as larger fluctuations in electric field gradients and increased molecular tumbling rates) overcoming effects of a larger population of sodium ions being incorporated into assemblies. Since the relaxation mechanisms of the sodium, in monomer or assembled state, are quite different from the mechanisms for phosphates (37), the overall effect of increased temperature on ^23^Na NMR could still be line narrowing but with different magnitudes of linewidths depending on whether phosphates are present to form assemblies.

### DOSY NMR.

**Fig. 4.**
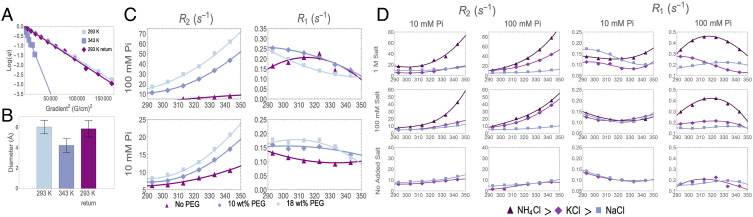
Evidence of entropically driven assembly. (*A*) 100 mM sodium phosphate at pH 4.2 ^31^P DOSY fits of Log(*ψ*) vs. gradient strength squared show a linear relationship, indicating that a single diffusion coefficient can describe behavior of phosphorous entities contributing to the observed NMR line. (*B*) Hydrodynamic diameters extracted from the diffusion coefficient from fits in (*A*). (*C*) *R*_2_ and *R*_1_ for potassium phosphate pH 4.5 in the presence of 6k MW polyethylene glycol (PEG) at varying PEG concentrations. Solid lines are quadratic fits to data to guide the eye. (*D*) *R*_2_ and *R*_1_ for orthophosphate pH 4.5 samples at 10 and 100 mM, with varying types of cationic salt chlorides and concentrations (see legend below). Salt-added samples are sodium phosphate salts, while no additional salt samples shown are monophosphate salts with the corresponding potassium or sodium cationic species. Solid lines are third-order polynomial fits to data to guide the eye. The *R*_2_ trends follow the predicted trends for the Hofmeister series, while *R*_1_ shows little difference at 10 mM phosphate concentration but significant differences for different salts at 100 mM.

Having established that larger phosphate assemblies exist in solution, we next explore the potential mechanisms of their assembly and, in particular, the temperature-dependent behavior. We performed pulsed field gradient (PFG) NMR, specifically diffusion-ordered spectroscopy (DOSY), to measure the self diffusion coefficients of the ^31^P NMR signal-bearing species and hence their hydrodynamic diameter. DOSY measurements were performed on a 100 mM sodium orthophosphate solution of pH 4.5 at 293 K and 343 K and again at 293 K after cooling in order to assess the reversible formation of any structures at elevated temperatures. The results show that the phosphate species in solution diffuse with a single translational diffusion coefficient, as demonstrated by the linear relationship between Log(*ψ*) and the square of the gradient strength, where *ψ* is the signal attenuated by molecular motion along the gradient axis (Fig. 4*A*) (38). This observation of a uniform diffusion coefficient did not change with increasing temperature. However, the diffusion coefficient significantly increased from 7.5  *  10^−10^ m^2^/s at 293 K to 3.2 * 10^−9^ m^2^/s at 343 K. We confirmed that this increase is not due to convection effects by comparing diffusion values measured with a convection-compensated pulse sequence (*SI Appendix*, Fig. S18) as well as in the presence and absence of capillaries added to the sample tube to disrupt convective flow (*SI Appendix*, Fig. S19) (39). To convert these diffusion coefficients to hydrodynamic diameters, we used the Stokes–Einstein relationship to account for the increased thermal energy and the decreased viscosity of water at elevated temperature. The extracted (temperature-corrected) hydrodynamic diameters for orthophosphate ions show a reversible and significant decrease by 1.8 Å at 343 K compared to 293 K (Fig. 4*B*), suggesting partial dehydration of hydrated orthophosphate ions in water. Similar increases in the diffusion coefficient and decreases in the hydrodynamic diameter were observed for monophosphate ions in 100 mM and 1 M potassium phosphate samples and 1 M sodium phosphate samples (*SI Appendix*, Figs. S20 and S21).

How do we reconcile the observation of assembly of orthophosphates according to ^31^P NMR relaxation and CEST studies with the apparent decrease in the hydrodynamic radius of orthophosphate molecules, implying the partial dehydration of the detected phosphate ions at elevated temperatures? Presumably, the partially dehydrated phosphate groups can more strongly interact with other phosphate groups through stronger hydrogen bond formation. Hence, they may more readily assemble into, and exchange with, dynamic phosphate clusters. It is known that a single deprotonated orthophosphate moiety, the H_2_PO${}_{4}^{-}$ ion, carries 11 water molecules within its hydration shell at infinite dilution (the HPO${}_{4}^{-2}$ ion carries 20 water molecules) (40). The ^31^P DOSY result for samples of pH 4.2, where the large majority of the population is in the H_2_PO${}_{4}^{-}$ protonation state, suggests a decrease in the hydrodynamic diameter from 6 Å to 4.2 Å. This change in hydrodynamic radius is consistent with a decrease in hydrodynamic volume by 70 Å^3^. Assuming a water radius of 1.4 Å (41), this result suggests a loss of 6 hydration water molecules upon heating, yielding a total of 5 remaining hydration water molecules per orthophosphate at 343 K. Given that the temperature-dependent characteristics of ^31^P NMR linewidth and relaxation data were observed across a wide pH range from 2 to 11, we expect both H_2_PO${}_{4}^{-}$ and HPO${}_{4}^{-2}$ ions to experience loss of hydration water with increasing temperature.

To further validate this experimental analysis based on DOSY, we performed MD simulations of less concentrated HPO_4_^2−^ and H_2_PO_4_^−^ systems discussed earlier. MD calculations show a decrease of water coordination from 13.4 to 13.1 per HPO_4_^2−^ molecule and from 16.5 to 15.7 per H_2_PO_4_^−^ with increasing temperature from 293 K to 343 K (*SI Appendix*, Fig. S15*C*), corresponding to the range of temperatures experimentally probed. These results are qualitatively consistent with the trends observed experimentally. Although this average change is relatively small, it is being driven by very large changes in hydration for the phosphates forming assemblies. Additionally, the decrease in the hydration number of orthophosphates is less dramatic in the MD simulations, as the computational analysis does not probe hydrodynamic radii but rather local density.

Is DOSY then detecting the phosphates within clusters directly? As discussed, phosphorus spins in these clusters undergo rapid relaxation due to their slower tumbling rates and thus have very broad resonance lines, largely invisible to ^31^P NMR. Thus, DOSY measurements should only be sensitive to the free phosphate ions that exist in equilibrium with these larger, spectroscopically dark, assemblies. The DOSY results reveal that free phosphate ions exchanging with the phosphate assemblies are more dehydrated at elevated temperatures and hence potentially have a greater tendency to assemble into larger clusters.

### Examining Entropy-Driven Assembly.

What then is the driving force for the formation of soluble, noncovalent, phosphate assemblies at equilibrium that are reversibly promoted at elevated temperature? Considering the Gibbs free energy for assembly, *ΔG*_*PA*_, an increasing tendency to assemble ($\Delta G_{\mathrm{PA}}<0$) at higher temperature requires that $\Delta S_{\mathrm{PA}}$ for assembly be positive, so that the entropic contribution lowers the free energy of assembly as temperature is increased, since generally ΔH increases (i.e., is less favorable) with increasing temperature (42). The thermodynamic parameters found in fitting our experimental *R*_2_ and CEST data of *ΔH*_*PA*_ = 25 kJ/mol and *ΔS*_*PA*_ = 30 J/(mol K) are consistent with an entropy-driven assembly. However, considering the fit value of *ΔH*_*PA*_ = 25 kJ/mol and a 0.6% population growth from 20 to 70 °C for a NaH_2_PO_4_ sample, the largest ΔH extracted would amount to only 0.002 J/°C/g solution for assembly formation of a 1 M potassium orthophosphate sample, rendering direct experimental verification of these thermodynamic values on our DSC instrument unfeasible. Nonetheless, an extensive set of experimental data provided strong evidence for a favorable phosphate assembly with increasing temperature, implying that the phosphate assembly is enhanced by entropy gain with increasing temperature.

Possible sources for this putative entropy gain are depletion interactions, including excluded volume effects, counterion release, and/or dehydration of the phosphate moiety (43). Excluded volume interactions between phosphates would not be expected to reduce the hydrodynamic diameters of individual phosphate monomers, and species with overlapping volume would codiffuse, resulting in slower diffusion, neither of which effects are observed by DOSY. Another commonly expected source of entropy gain upon assembly of charged species is the release of bound counterions; however, potassium and sodium ions are not strongly bound to phosphate, making its release a less likely source for significant entropy increase. This assessment is consistent with our MD simulation results, which show that there is no significant change in the number of counterions coordinated with HPO_4_^−2^ and a slight increase in the counterion coordination number for H_2_PO_4_^−^ in the less concentrated (∼78 mM) systems (*SI Appendix*, Fig. S15*I*). This analysis further supports our expectation that the phosphate–cation interaction is weak, making counterion release an unlikely driver of phosphate assembly. Furthermore, the observed changes in the hydrodynamic diameter of orthophosphates with increasing temperature as measured by DOSY are very similar between potassium and sodium phosphate samples (Fig. 4*A* and *SI Appendix*, Fig. S21). Since sodium and potassium ions are approximately 1 Å different in size (44), a difference in the change of the hydrodynamic diameter is expected if counterion release was a major contributor to these observed size changes of orthophosphates.

Hence, the most likely source of increase in the total entropy is the shedding of water that is more strongly associated with the phosphate ions than with bulk water, also referred to as the hydration shell. Water forms networked hydrogen bonds and strongly solvates phosphate anions, offering ample opportunities for entropy increase upon its partial release. DOSY experiments and MD simulations confirm that water molecules are released to bulk when increasing the temperature from 293 K to 343 K. In a recent study, dehydration-driven entropy increase has been shown to be a primary driver of liquid–liquid phase separation induced by polyelectrolyte assembly processes in water (45).

### Manipulation of Depletion Interactions.

Since the experimental results so far suggest dehydration entropy as the driver of phosphate assembly, we designed further experiments to deliberately modulate the phosphate–water interactions by known factors. Since we established a viable interpretation for the change in ^31^P NMR linewidth and relaxation data, we rely on these readouts to evaluate phosphate assembly formation as a function of temperature.

One can amplify dehydration by the addition of hydrophilic molecular crowders or salting-out salts along the Hofmeister series. The introduction of molecular crowders is a common technique used to reduce the volume of water available for the other molecules of interest in an aqueous solution, thus increasing the effective concentration of the dissolved molecule (45). A commonly used molecular crowder is polyethylene glycol (PEG); its strong affinity for water over the temperature range of interest is known to drive dehydration and increase the effective concentration of other molecules in solution (46). As expected, the ^31^P NMR linewidth at temperatures ranging from 293 K to 343 K increased for phosphate solutions at both 10 mM and 100 mM concentrations with increasing PEG concentrations at 10 wt% and 18 wt%. In order to evaluate whether these observed trends are due to a contrast in solution viscosity, *R*_2_ relaxation extracted from FWHM for two samples of 100 mM potassium phosphate, with and without 18 wt% PEG, was plotted as a function of solution viscosity (*SI Appendix*, Fig. S22). The *R*_2_ relaxation rates of the two samples are not superimposed, indicating that the observed increase in linewidth is not accounted for solely by changes in solution viscosity from the addition of PEG. This observation is consistent with the interpretation that PEG enhances the dehydration of phosphates and facilitates phosphate clustering.

Dehydration can also be modulated by the addition of various salts according to the Hofmeister series (47). This series is used in biological systems to induce salting-out (precipitation) or salting-in (dissolution) of proteins, with NH${}_{4}^{+}$ on one salting-out end and Na^+^ on the other salting-in end (48) of the series. While phosphate clusters under the here tested experimental conditions are not precipitated out of solution, the magnitude of dehydration, and thus the exchange with and/or formation of assemblies, should increase with salting-out salts and decrease with salting-in salts. To test our hypothesis of dehydration-driven phosphate clustering, we added a variety of cations that enhance the salting-out tendency in the order NH${}_{4}^{+}$ >K^+^ >Na^+^. ^31^P NMR linewidths were measured between 293 K and 343 K for orthophosphate solutions at 10 mM and 100 mM in the presence of added chlorine salt of three different cations at 100 mM and 1 M concentrations, as well as in the absence of added salts. The extracted linewidths show that the addition of NH${}_{4}$^+^ causes the greatest line broadening, followed by K^+^ and then Na^+^, at all phosphate concentrations, salt concentrations, and temperatures (Fig. 4*D*). These results are in agreement with the predicted trend of the Hofmeister series when considering line broadening as a proxy for dehydration-induced clustering. While the *R*_1_ results are more difficult to interpret, given the nonmonotonic trend, we see that the salts clearly change the shape and magnitude of *R*_1_ when phosphate and/or salt concentration is high enough (Fig. 4*D*). This finding is again in agreement with the hypothesis that the addition of salting-out salts impacts the tendency of phosphates to form assemblies.

Molecular crowders and salting-out cations both serve to increase the total entropy of dehydration, further facilitated by elevated temperatures, consistent with the ^31^P NMR results. While the chemical exchange between phosphate species of different protonation states or scalar relaxation may potentially explain some of the observed anomalous ^31^P NMR line broadening behavior with increasing temperature, these alternative hypotheses do not provide comprehensive explanations for the full range of results provided here. The proton exchange rate for a 100 mM phosphate sample is 10^7^ Hz, many orders of magnitude larger than the strength of proton–phosphorus scalar couplings, indicating that scalar relaxation does not dominate. Similarly, a model of chemical exchange between differently protonated phosphate groups cannot explain the order of magnitude discrepancy between *R*_1_ and *R*_2_. This order of magnitude difference between *R*_1_ and *R*_2_ also indicates that paramagnetic impurities could not be causing the enhanced relaxation; as for phosphate and paramagnetic species tumbling freely in solution, one would expect *R*_1_ and *R*_2_ to be close to identical. Additionally, these potential explanations fail to explain the other results shown, such as the broad CEST lines, the cryo-TEM images, or the multiple experiments indicating that phosphate dehydration plays a role in modulating ^31^P NMR relaxation properties.

## Conclusions

Taken together, our experiments present comprehensive evidence for the presence of phosphate assemblies in aqueous solutions in dynamic exchange with free phosphate. ^31^P NMR relaxation and CEST results both show signatures of the presence of these phosphate assemblies, whose population grows with increasing temperature. Cryo-TEM offers visual affirmation for the presence of large phosphate assemblies in both monophosphate and ADP solutions, with structures consistent with condensed spherical droplets. ^31^P DOSY measurements, as well as the impact of PEG and cationic salts, indicate that dehydration of phosphate species is driving the formation of these assemblies. Even if the number of water molecules released per orthophosphate molecule was small, its effect on entropy gain driving the assembly formation can be very significant, given that the assembly consists of a large number of orthophosphates according to our model derived from R_2_ and CEST data, cryo-TEM data, and MD simulations. Our results suggest that this dehydration-driven clustering of phosphates may be present in numerous species with exposed phosphate groups at a wide range of commonly used solution conditions, including those of biological relevance. Given these findings, and the ubiquity of phosphates in biological systems, we propose that such clusters should be considered in the interpretation of both in vivo and in vitro experiments involving phosphate group-containing biomolecules.

## Materials and Methods

Potassium phosphate monobasic (MW 136.09) and sodium phosphate monobasic (MW 119.98) were acquired from Fisher Scientific. Sodium phosphate tribasic (MW 163.94) was acquired from Acros Organics. Potassium pyrophosphate (MW 330.34) was acquired from Sigma-Aldrich. Adenosine 5’-diphosphate orthopotassium salt dihyrdate (MW 501.32) was acquired from Alfa Aesar. Adenosine 5’-triphosphate disodium salt hydrate (MW 551.14 anhydrous) was obtained from Sigma. Polyethylene glycol (MW 6K) was acquired from Fluka. Potassium chloride (MW 74.55), sodium chloride (MW 58.44), and ammonium chloride (MW 53.49) were acquired from Fisher Chemical. All samples prepared at room temperature. When not explicitly mentioned, the pH values were adjusted to 4.4 with HCl and NaOH to coincide with the native dissolved pH values found for monobasic orthophosphate. Every sample was dissolved in 600 to 700 μL of 90% Milli-Q water and 10% D_2_O for locking purposes.

Adenosine 5’-diphosphate sodium salt (MW 427.20), sodium phosphate dibasic (MW 141.96), and coenzyme A sodium salt hydrate (MW 767.53) used for CEST were acquired from Sigma-Aldrich. All samples were prepared at room temperature. The pH values of the solutions were adjusted to 4.4 with HCl and NaOH in order to make them coincide with the native dissolved pH values found for orthophosphate. The real concentrations of the solutions were determined from the absolute integrations of the ^31^P peaks in the 1D NMR spectra.

### NMR Experiments.

Solution NMR relaxation experiments were performed on a Bruker Avance NEO 500 MHz spectrometer with a CryoProbe Prodigy BBO probe, using Wilmad-LabGlass 5-mm Thin Wall Precision NMR tubes. T_1_ relaxation was measured with a standard inversion-recovery pulse sequence, and T_2_ relaxation was measured using a CPMG sequence. Delays varied depending on sample conditions [temperature, pH, and concentrations of salts, and polyethlyene glycol (PEG)].

T_1_ and T_2_ delays were experimentally modulated such that the final two points for T_1_ curves fully recovered and the final point for T_2_ curves was less than 5% of the initial intensity. T_1_ relaxation times were determined by employing the TopSpin 4.0.6 T1/T2 dynamics module. T_2_ relaxation times were determined by MestReNova monoexponential fitting. FWHM was determined by taking a 45° pulse and employing TopSpin 4.0.6 PEAKW command. For each spectrum, a single scan was acquired with 40,000 data points to cover a spectral window of 10,000 Hz (49.4 ppm). An AU program was created to ensure temperature equalization uniformity which included a 10-min temperature equilibration time, and autoshimming was applied continuously before and throughout acquisition.

DOSY measurements were taken on a 300 MHz SWB Bruker spectrometer with a single gradient along the z-axis. DOSY is an experiment that uses the pulsed field gradient NMR (PFG-NMR) technique to extract diffusion coefficients for each NMR signal present in a sample. PFG-NMR measures particle diffusion by using a spin-echo pulse sequence in combination with a magnetic field gradient. As particles diffuse during the spin-echo sequence, they experience a slightly different field due to the gradient, and the spin-echo is unable to completely rephase the signal. This dephasing causes an attenuated signal intensity, which depends on the strength of the gradient, *g*, and the diffusion coefficient, *D* of the species as $\psi \left(g,D\right)=Exp(-Dg^{2}\gamma^{2}\delta^{2}\left(\Delta -\delta /3\right))$, where *γ* is the gyromagnetic ratio of the nucleus, *δ* is the width of the gradient pulse, and *Δ* is the time between gradient pulses (38). By measuring this signal attenuation at several gradient strengths, one is able to fit the attenuation function to recover the diffusion coefficient of the associated species at each NMR line.

Measurements were taken at 293 K and 343 K and again at 293 K after the sample had cooled. Capillaries were used at elevated temperatures to suppress convection. To confirm that there were not convection effects, a convection-compensated pulse sequence was compared to the standard sequence for one sample at 293 K and 343 K. At 293 K, 32 scans with a 90° pulse were acquired with 16384 data points to cover a spectral window of 6,068 Hz (49.9 ppm) for gradient strength, and at 343 K, 64 scans with a 90° pulse were acquired for each gradient strength with the same spectral conditions as above. At each temperature, data were taken using 16 linearly spaced gradient strengths such that the final spectrum had an intensity less than 5% of the first’s. These decays were then fit in the TopSpin 4.0.6 T1/T2 relaxation module to extract a diffusion coefficient. The Stokes–Einstein relation was then used to convert this to a hydrodynamic diameter.

NMR CEST experiments were performed on a Bruker 500 MHz (11.7 T) NMR spectrometer equipped with a broadband observe (BBO) probe. The 90° pulse duration ranged from 10 to 12 us depending on ionic strength of the solution. Saturation was performed by continuous wave (cw) irradiation of 5-s duration with field strengths of 1.16 μT to 8.69 μT (corresponding to nutation frequencies of 20 Hz to 150 Hz). The recycling delay was set to 5 s. Following cw irradiation, a 90° pulse is used for spectral readout. The temperature dependence of CEST measurements was taken with the irradiation power of 150 Hz at 298 K, 313 K, 323 K, 333 K, 343 K, and 353 K, and the irradiation power dependence of CEST measurements was taken at 298 K with nutation frequencies of 20 Hz, 30 Hz, 50 Hz, 100Hz, and 150 Hz. At each measurement, eight scans with a 90° pulse were acquired. The scanned frequency ranged from −4,000 Hz to 4,000 Hz with a step size of 200 Hz.

Control experiments were performed to rule out the influence of temperature gradients or convection on the results. Relaxation rates were found to be the same in samples containing capillaries (used to curb convection, if it exists), and chemical shift imaging was used to verify that the linewidths were the same in different z-positions in the sample.

### Cryo-TEM Experiments.

Phosphate solutions were prepared at room temperature and heated for at least 48 h at 343 K before vitrification. Solutions were vitrified using an FEI Vitrobot Mark IV for vitrification in liquid ethane. On a lacey carbon support film on a 200-mesh copper grid, 1.2 μL of the sample was deposited, blotted for 1 s, and immediately vitrified. The cryo-TEM was performed using a Gatan 626 Cryo transfer holder with liquid nitrogen by ThermoFisher Talos G2 200X TEM/STEM at 200 kV with a Ceta II CMOS camera for bright-field imaging.

## Supplementary Material

Appendix 01 (PDF)

## Data Availability

All study data are included in the article and/or *SI Appendix*.
