# Supplementary material for: Phosphates form spectroscopically dark state assemblies in common aqueous solutions
Source: Proc Natl Acad Sci U S A. 2022 Dec 29;120(1):e2206765120. doi: 10.1073/pnas.2206765120 (PMC9910612; doi:10.1073/pnas.2206765120)
Supplement: Supplementary file 1 — Appendix 01 (PDF) [file pnas.2206765120.sapp.pdf]

Supplementary Information for

Phosphates form spectroscopically dark state assemblies in common aqueous solutions

Joshua S. Straub, Mesopotamia Nowotarski, Jiaqi Lu, Sally Jiao, Tanvi Sheth, Matthew P.A. Fisher, M. Scott Shell, Matthew E. Helgeson, Alexej Jerschow, Songi Han

[2]E-mail: songihan@ucsb.edu, alexej.jerschow@nyu.edu

This PDF file includes:

Supplementary text
Figs. S1 to S22 (not allowed for Brief Reports)
Table S1 (not allowed for Brief Reports)
SI References

Supporting Information Text

Figures

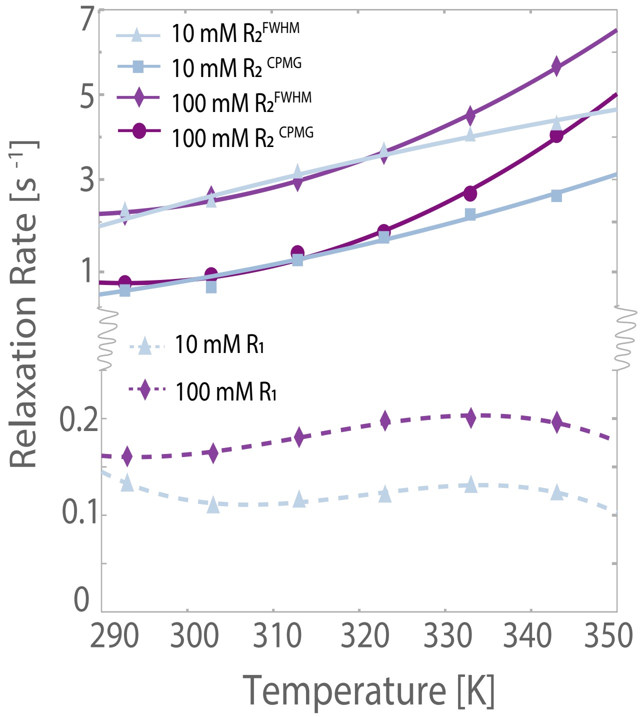

Fig. S1. R_2 as extracted from a CPMG pulse sequence and from FWHM for 10 mM and 100 mM potassium orthophosphate monobasic pH 4.5 as a function of temperature, showing monotonic increase in R_2 in each case. Solid lines are quadratic fits to data to guide the eye. R_1 for 10 mM, 100 mM, potassium orthophosphate monobasic pH 4.5 as a function of temperature showing the same curve shapes as a function of concentration. Solid lines are cubic fits to data to guide the eye.

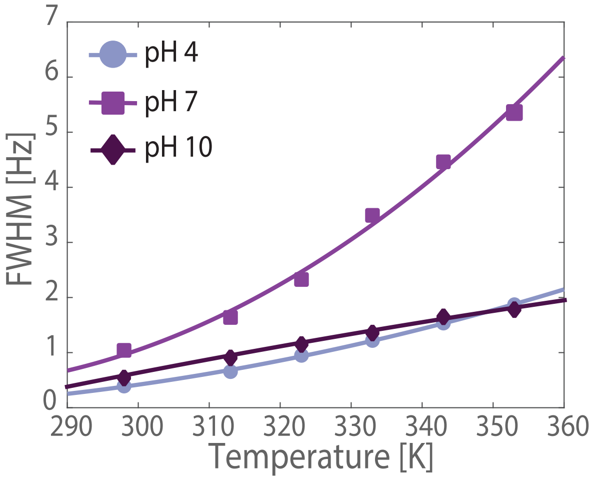

Fig. S2. FWHM of sodium phosphate dibasic solutions at pH 4, pH 7 and pH 10 showing line broadening. Solid lines are quadratic fits to data to guide the eye.

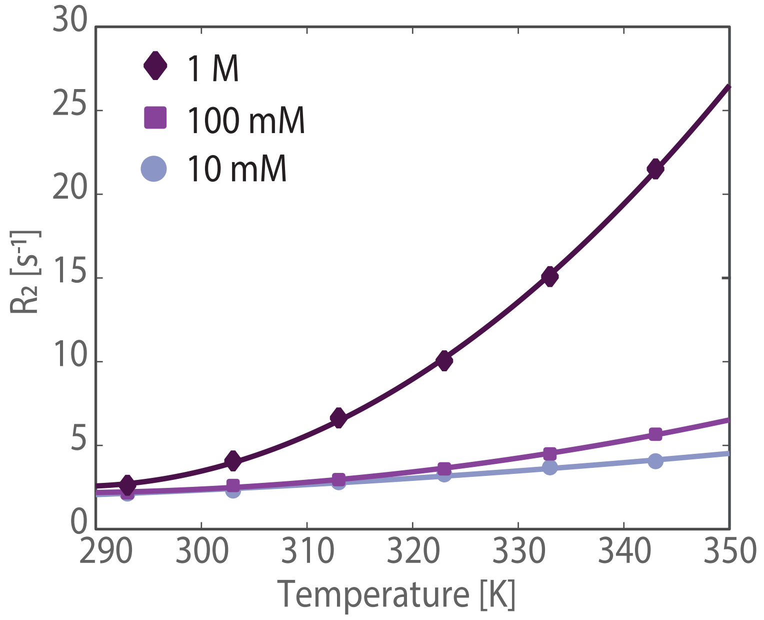

Fig. S3. R_2 rates calculated from FWHM for potassium phosphate solutions at pH 4.5 over a range of phosphate concentrations showing line broadening across a range of concentrations. Solid lines are quadratic fits to data to guide the eye.

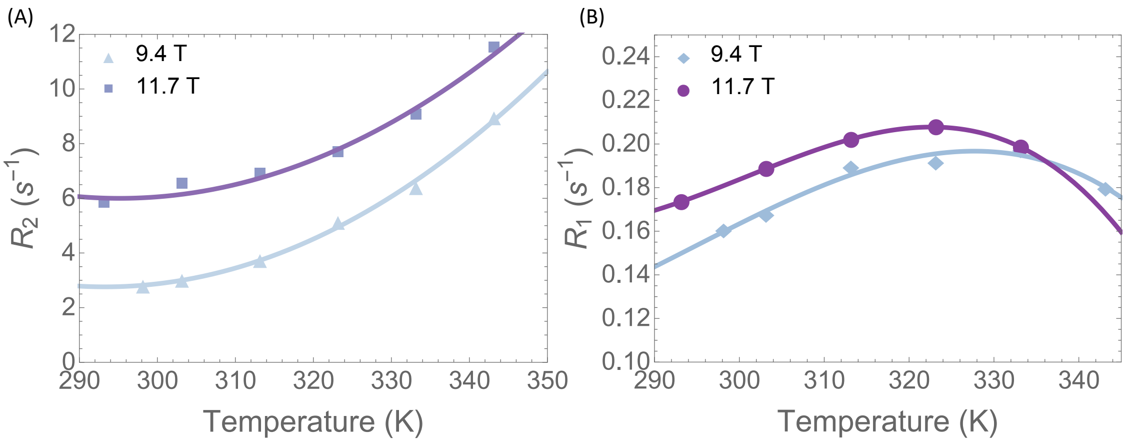

Fig. S4. Relaxation rates for 10 mM potassium phosphate at pH 4.5 from two different spectrometer strengths- 9.4 T ($\omega_0(^{31}P)$ =162 MHz) and 11.7 T ($\omega_0(^{31}P)$=202 MHz. (A) R_2 as calculated from FWHM shows increase with temperature at both spectrometer strengths.Solid lines are quadratic fits to data to guide the eye. (B) R_1 rate minimums shift to higher temperatures at greater field strength.Solid lines are third order polynomial fits to data to guide the eye.

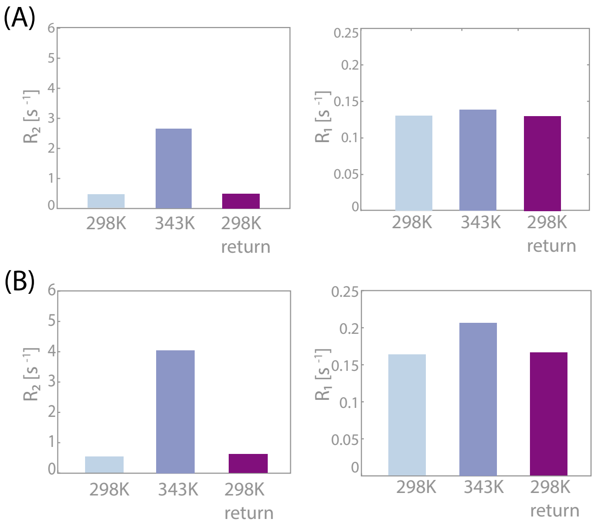

Fig. S5. Relaxation Rates for 10 mM and 100 mM sodium phosphate show reversibility after heating to 343K and cooling. (A) Relaxation rates for 10 mM sodium phosphate, (B) Relaxation rates for 100 mM dosium phosphate

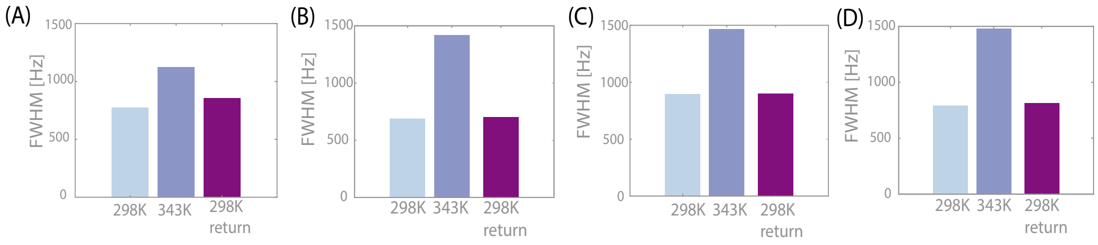

Fig. S6. ^{31}P CEST dip widths showing Reversibility at 298 K, 353 K and cooling back to 298 K. (A) 50 mM orthophosphate, (B) 100 mM orthophosphate, (C) 200 mM orthophosphate, (D) 300 mM orthophosphate.

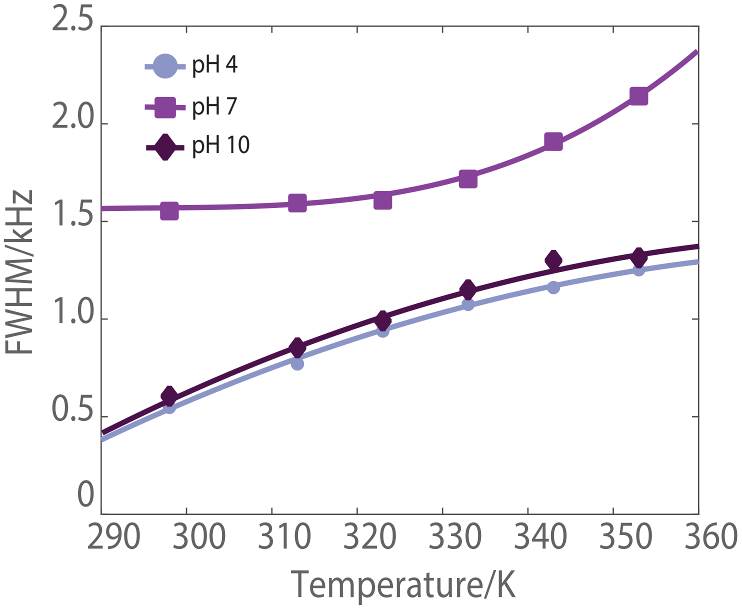

Fig. S7. ^{31}P CEST dip widths at half height of sodium phosphate dibasic solutions at pH 4, pH 7 and pH 10 showing line broadening as a function of temperature. Solid lines are quadratic fits to data to guide the eye.

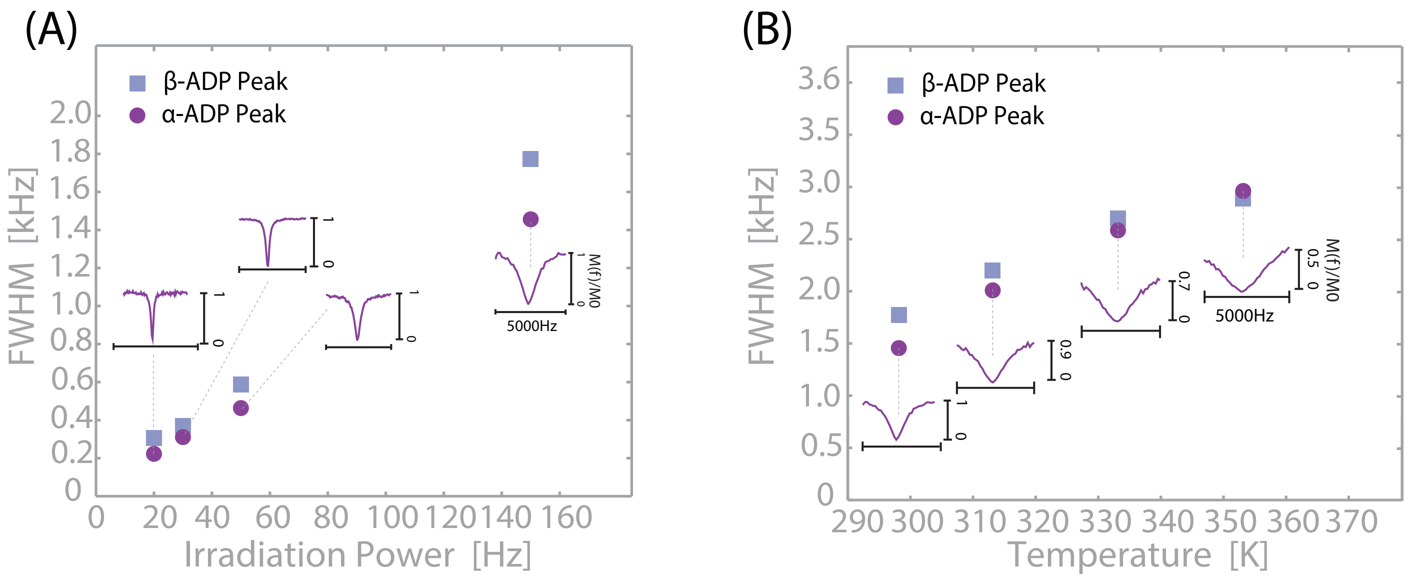

Fig. S8. ^{31}P CEST results for both phosphates in 100 mM ADP in D_2O at pH 3.9. (A) CEST dip width at half height as a function of CEST irradiation power of 20 Hz, 30 Hz, 50 Hz and 150 Hz at T=298.15 K, (B) CEST dip width at half height as a function of temperature of 298 K, 313 K, 333 K, 353 K.

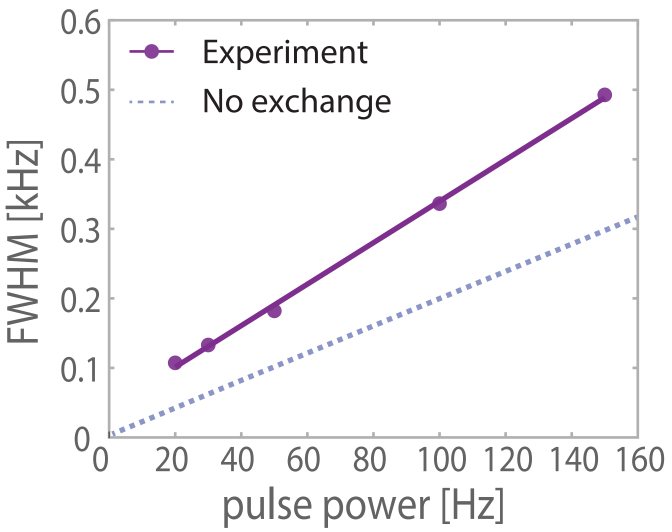

Fig. S9. ^{31}P CEST dip width at half height for 100 mM sodium phosphate as a function of CEST irradiation power of 20 Hz, 30Hz, 50 Hz, 100 Hz, and 150 Hz measured at 298 K. In the absence of exchange, one expects the width of the dip in the CEST spectrum to be approximately a factor two larger than the rf saturation power (expressed in Hz). However, the dip width ranging from 100 Hz to 500 Hz under exchange is over a factor three larger than the rf saturation power (expressed in Hz). The Solid lines are linear fits to guide the eye

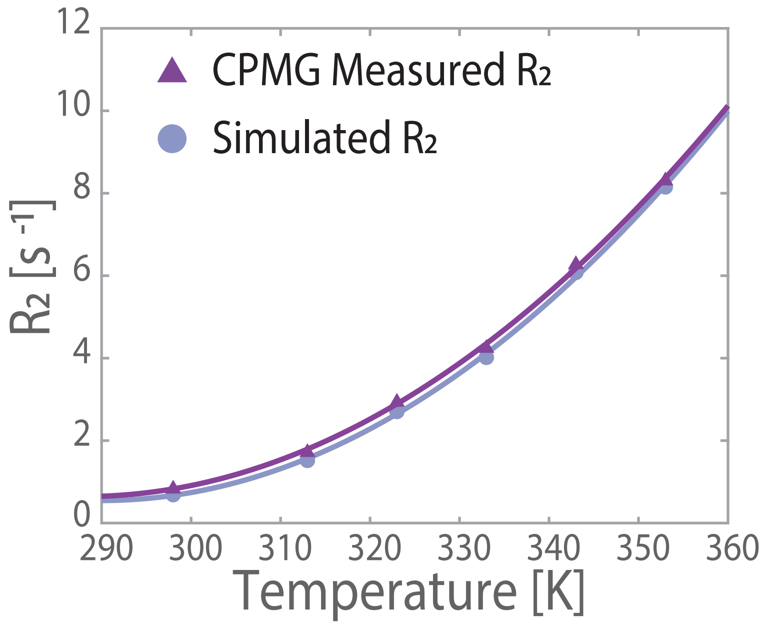

Fig. S10. The experimental and simulated ^{31}P R_2 rates for the 100 mM orthophosphate sample shown in Figure 2*B*&*C*. Parameters used for this simulation were the same as used for the CEST simulations in Figure 2B. Additional information on the modeling and physical interpretation is below.

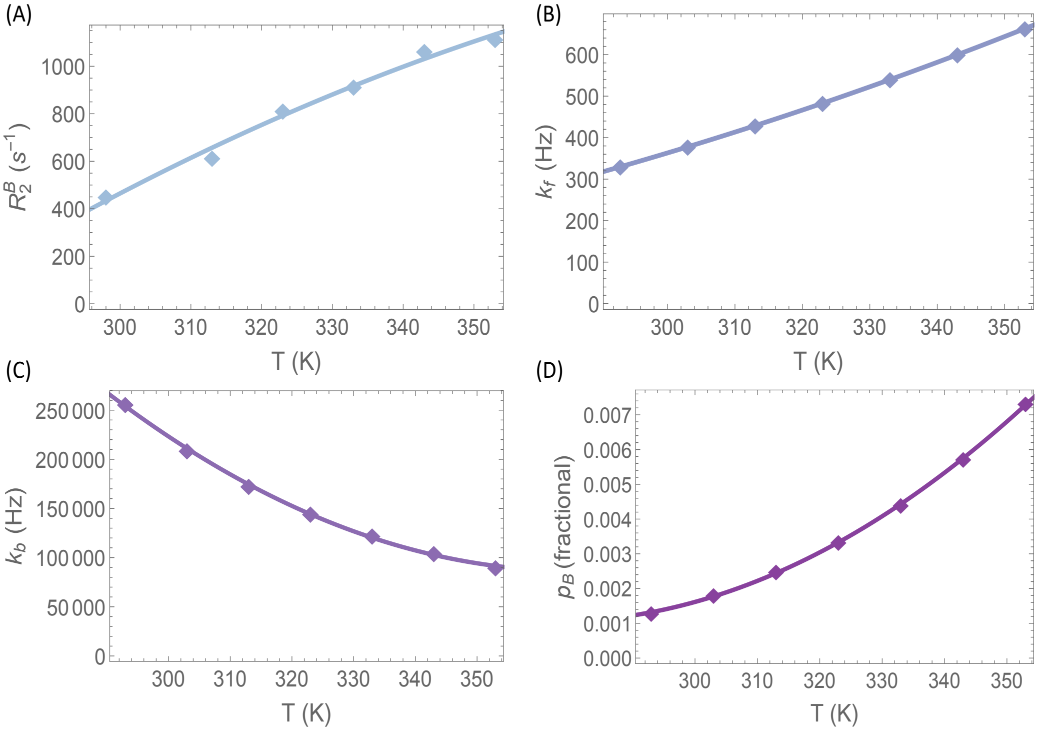

Fig. S11. Temperature dependence of R_2^B, kinetic parameters, and assembly population used for the simulated R_2 and CEST data shown in Figure S10 and 2*B*, respectively. Solid lines are quadratic fits to data to guide the eye. (A) The assembly relaxation rate R_2^B increases from 450 Hz to 1100 Hz between 298 K and 353 K. (B) The phosphate monomer to assembly rate (k_f) increases from 350 Hz to 600 Hz between 293 and 353 K. (C) The assembly to monomer rate (k_b) decreases from 250,000 Hz to 100,000 Hz between 293 and 353 K. (D) The fractional assembly population (p_B) increases from 0.0013 to 0.0073 between 293 K and 353 K.

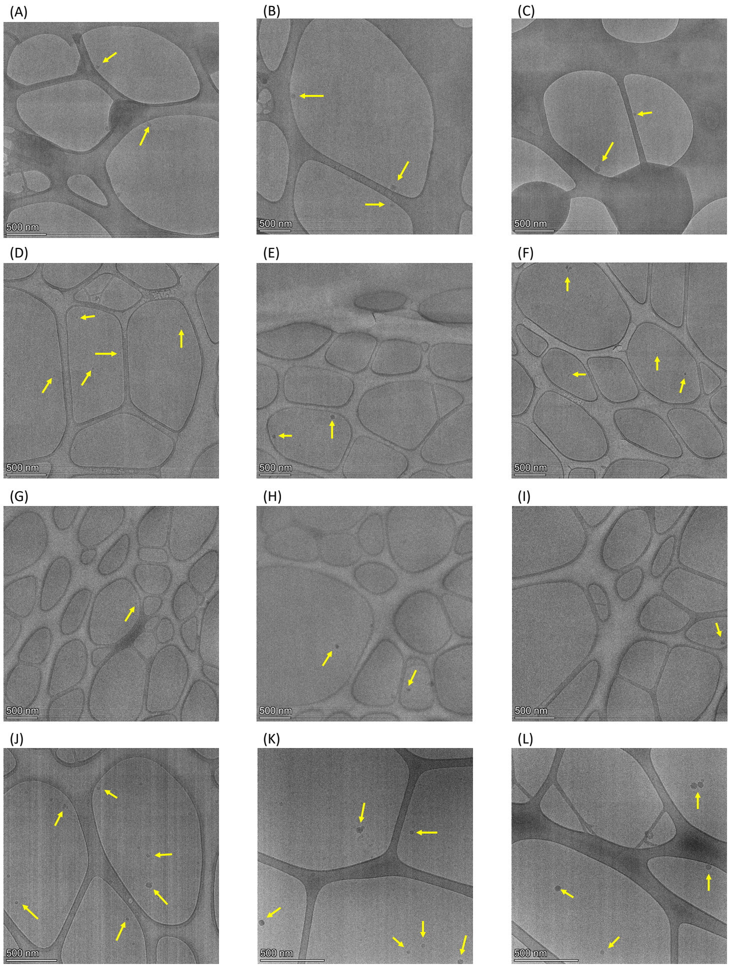

Fig. S12. Cryo-TEM micrographs from (A)-(C) 100 mM sodium ADP sample unheated, (D)-(F) 100 mM sodium ADP heated to 343 K, (G)-(I) 500 mM KCl heated to 343 K, and (J)-(L) 100 mM potassium ADP heated to 343 K. Yellow arrows indicate features in the size range of assemblies, although ice artifacts can appear at similar sizes. Samples in (A)-(I) are the samples used for the quantitative particle analysis.

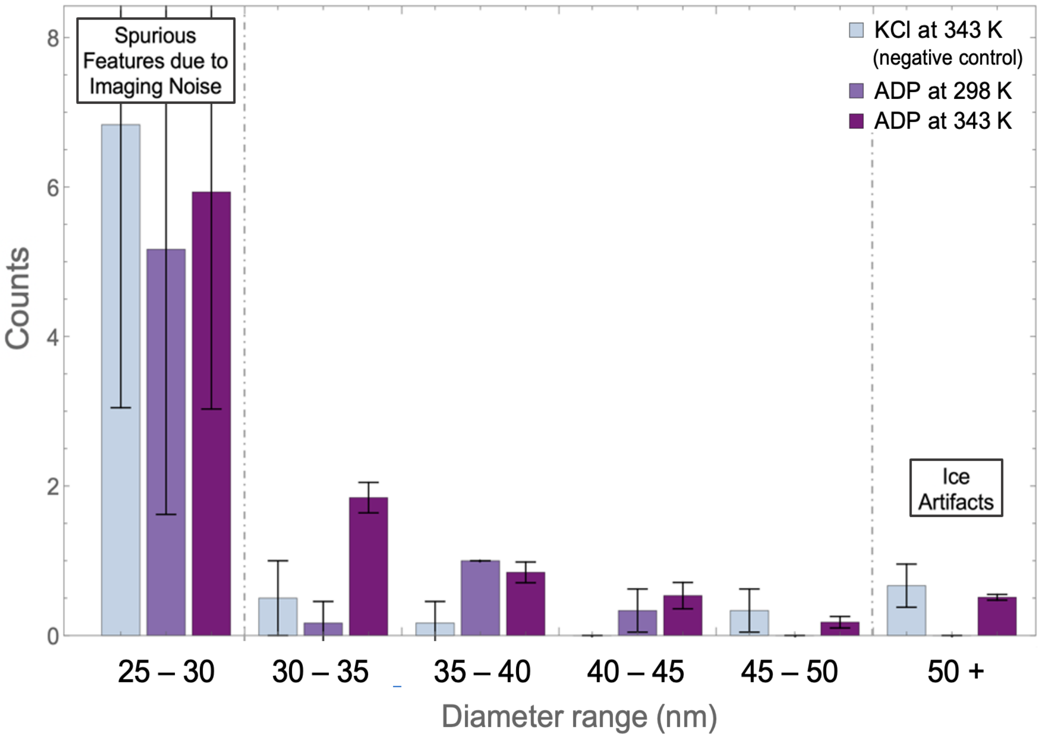

Fig. S13. Histogram of diameters for features detected per micrograph in selection of representative micrographs for sodium ADP at 298 K, sodium ADP heated at 343 K, and potassium chloride heated at 343 K. Only micrographs taken at the same magnification were compared, since the micrograph noise is primarily on pixel level. There were two, nine, and two micrographs, respectively, thus analyzed in the workflow described in SI. These results show a larger population of species in the two ADP samples, with the most in the heated ADP samples, except at the smallest and largest sizes, which can thus be attributed to erroneous features from the processing of the image analysis and ice artifacts, respectively, given that these are the only populations seen in the potassium chloride samples.

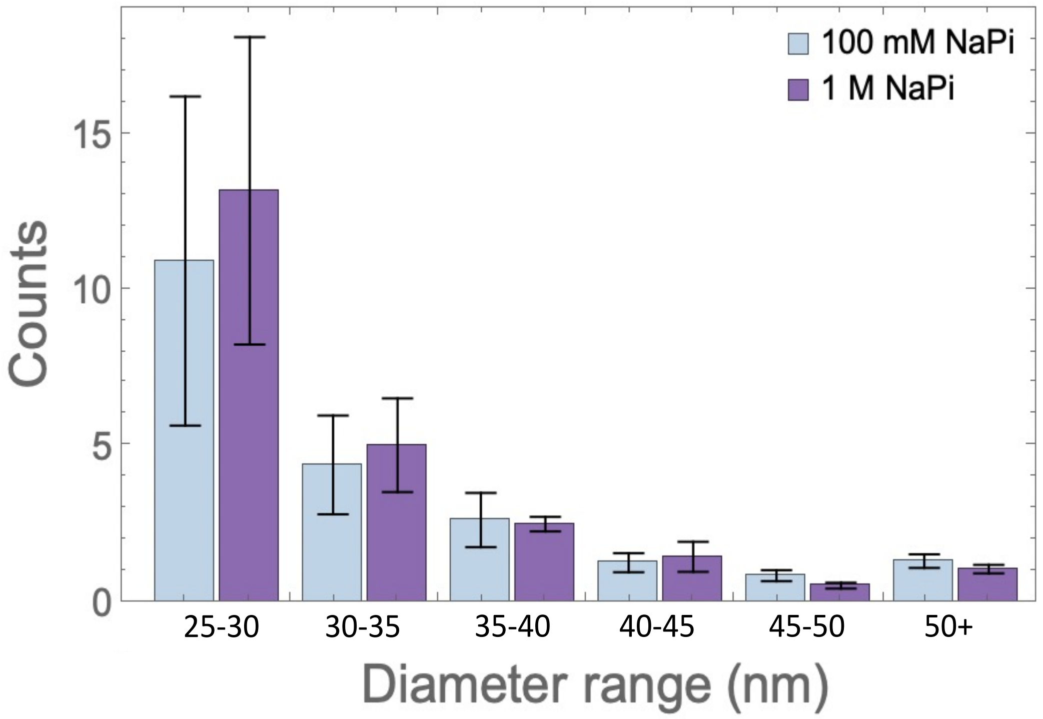

Fig. S14. Histogram of diameters for features detected per micrograph in selection of representative micrographs for sodium monophosphate at 100 mM and 1 M heated to 343 K before vitrification. Only micrographs taken at the same magnification were compared, since the micrograph noise is primarily on pixel level. Each sample incorporates data from 20 micrographs analyzed with the workflow described in SI. These results show that the size distribution of features is similar at both concentrations, which is consistent with equilibrium structures.

Cryo-TEM Image processing workflow

Microscopy analysis was conducted in Fiji Version 1.0. Images were first processed with a FFT bandpass filter, in order to smooth long-length scale variations in intensity to to variations in camera pixel sensitivity, and to attenuate structures at small length scales that originate from pixel noise. Cutoffs for these filtering were varied, and found not to qualitatively affect the relative results. After filtering, pixels were autoscaled and saturated, before thresholding on the top 2-4 % of pixel intensity. The threshold was varied in this range to provide error bars for histogram results. After thresholding, the Close function was used to fill small regions of white pixels fully surrounded by black pixels. The Analyze Particles function was then used to select particles with an area of $200 - 2000$ pixels^2 and a circularity criteria of $0.6 - 1$, where circularity is defined as $4\pi * \frac{area}{perimeter^2}$. Results were then extracted and converted into area in nanometers for comparison between samples.

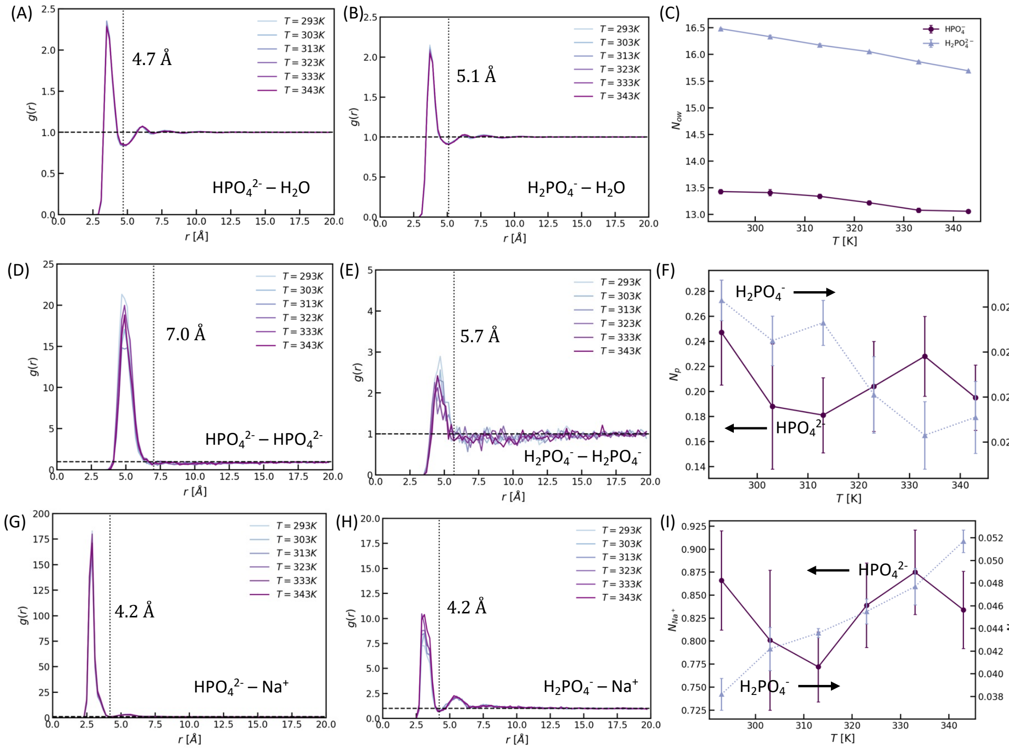

Fig. S15. Radial distribution functions (RDFs) and coordination numbers for water around phosphate ions, phosphate ions with each other, and Na^+ around phosphate ions. RDFs (*A*, *B*, *D*, *E*, *G*, *H*) are computed to a maximum distance of 20 Å with a bin size of 0.2 Å. The distances r are between (*A*, *B*) water oxygen atoms and phosphate phosphorous atoms, (*D*, *E*) phosphate phosphorous atoms with each other, and (*G*, *H*) sodium atoms and phosphate phosphorous atoms. The water-phosphate coordination number (*C*) is the average number of water oxygen atoms within 4.7 Å (HPO_4^{2-}) or 5.1 Å ($H_2PO_4^-$) of a phosphate phosphorous atom. The phosphate-phosphate coordination number (*F*) is the average number of phosphorous atoms within 7.0 Å (HPO_4^{2-}) or 5.7 Å ($H_2PO_4^-$) of a phosphorous atom. The sodium-phosphate coordination number (*I*) is the average number of sodium atoms within 4.2 Å of a phosphate phosphorous atom. The coordination number cutoffs are determined from the approximate location of the first minimum of the corresponding RDF (dotted lines, labeled with the exact cutoff). Error bars in (*C*), (*F*), and (*I*) are twice the standard error of the coordination numbers computed for each 200-ns block in the 1000-ns production simulation.

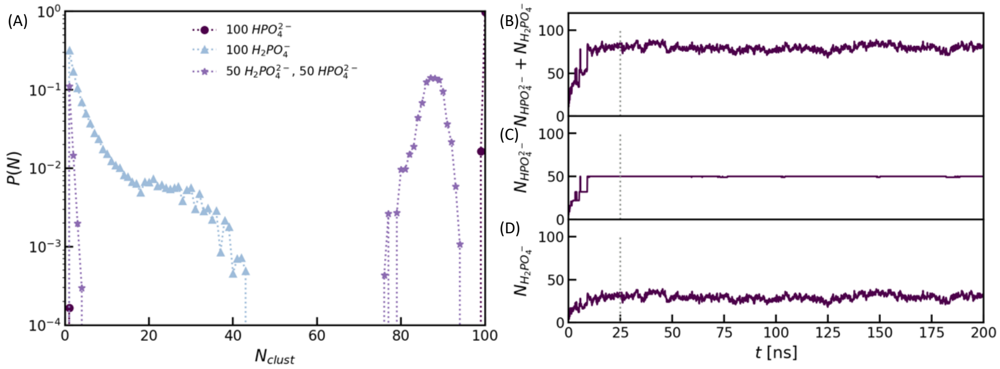

Fig. S16. (*A*) Cluster size distributions for the same systems as in Fig. 3*C* of the main text, but at 293 K. (*B*) Trajectory of the combined number of phosphate ions, both HPO_4^{2-} and $H_2PO_4^-$, in the largest cluster of the simulation of the mixed system at 343 K during the NPT equilibration and NPT production (separated by the dotted line). (*C*) Trajectory of the number of HPO_4^{2-} ions in that largest cluster. This trajectory is virtually constant at $N = 50$, the total number of HPO_4^{2-} ions in the box, during the entire production run, indicating that all HPO_4^{2-} ions in the box are in this large cluster. (*D*) Trajectory of the number of $H_2PO_4^-$ ions in that same cluster. Two phosphate ions are considered clustered if their phosphorous atoms are within 7.0 Å of each other. The 7.0 Å cutoff is chosen from the approximate location of the first minimum in the phosphate-phosphate RDF (Fig. S13*C*).

MD simulation workflow and systems simulated

Systems are constructed using GROMACS 2016.1 (1, 2). We perform the simulations using the OpenMM simulation engine (3). Electrostatic interactions are computed using particle-mesh Ewald. Lennard-Jones nonbonded interactions are cut off at 10 Å. Hydrogen bonds are constrained with SHAKE (4) and water is kept rigid with SETTLE (5). We use Langevin dynamics with an integration timestep of 2 fs and a friction coefficient of 0.1 ps^{-1}. We first perform a local energy minimization, followed by a 100-ps NVT equilibration. For the concentrated systems, we then perform a 25-ns NPT equilibration using a Monte Carlo (MC) barostat with 200 fs between MC moves and then perform a 175-ns NPT production, again using the MC barostat with the same conditions, saving configurations every 100 ps. For the dilute systems, we perform a 100-ps NPT equilibration using the MC barostat with the same conditions, followed by a 1000-ns NPT production, again using the MC barostat with the same conditions. The cluster size distributions, radial distribution functions, and coordination numbers are computed from the production simulations.

$N_{HPO_4^{2-}}$	$N_{H_2PO4^-}$	N_{Na^+}	N_{H_2O}	Temperatures simulated [K]
100	0	200	4759	293, 343
0	100	100	4852	293, 343
50	50	150	4801	293, 343
3	0	6	2149	293, 303, 313, 323, 333, 343
0	3	3	2148	293, 303, 313, 323, 333, 343

Table S1. Systems simulated using molecular dynamics.

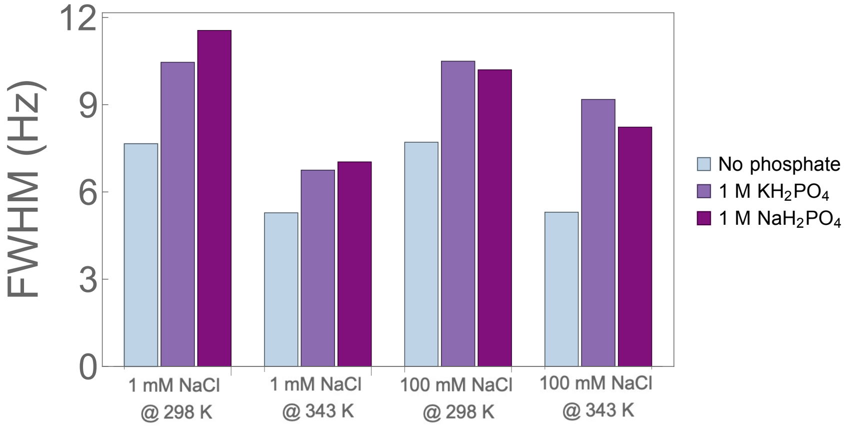

Fig. S17. Experimental ^{23}Na linewidth data for 1 mM and 100 mM NaCl in the absence of phosphates, and with 1 M KH_2PO_4 and 1 M NaH_2PO_4 at 298 K and 343 K. All solutions were adjusted to pH 4.1 with HCl. ^{23}Na linewidths are broader in the presence of phosphates at both temperatures and concentrations, indicating that sodium is incorporated into the assemblies.

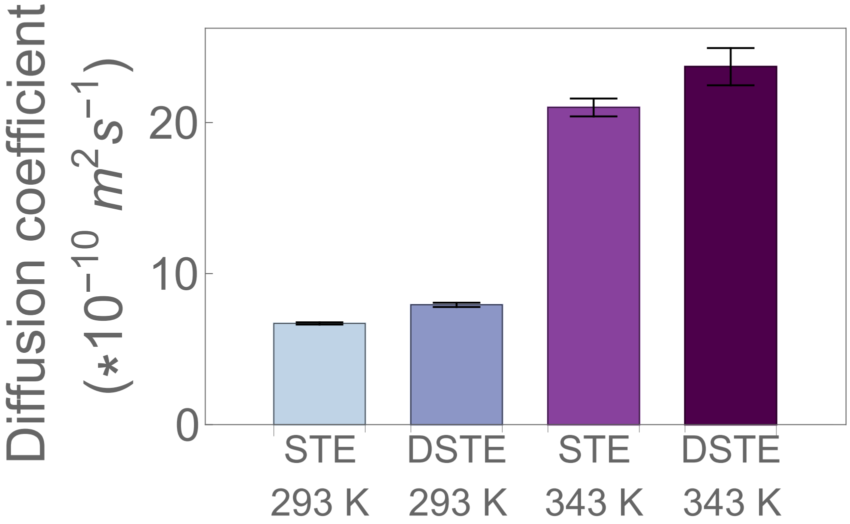

Fig. S18. Diffusion coefficients extracted from ^{31}P DOSY experiments for 1 M potassium phosphate at pH 4.2 using a stimulated echo (STE) and convection-compensated double stimulated echo (DSTE) sequence. Diffusion coefficients were found to be comparible at both 293 K and 343 K, indicating that the observed increased in diffusion coefficient with temperature is not a convection-based effect.

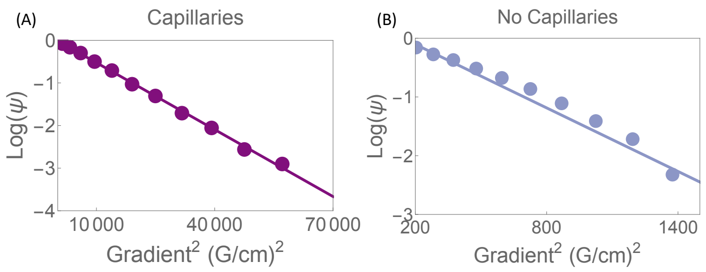

Fig. S19. ^{31}P DOSY fits of Log(ψ) vs gradient strength squared for a 1 M potassium phosphate sample in the presence (A) and absence (B) of capillaries at 343 K, where ψ is signal attenuation. Note that in the absence of capillaries, the relationship between Log(ψ) and gradient squared is non-linear due to convection-based dephasing effects, and that the gradient values are much lower because of the increased rate of particle motion. Capillaries were thus used for all high temperature samples.

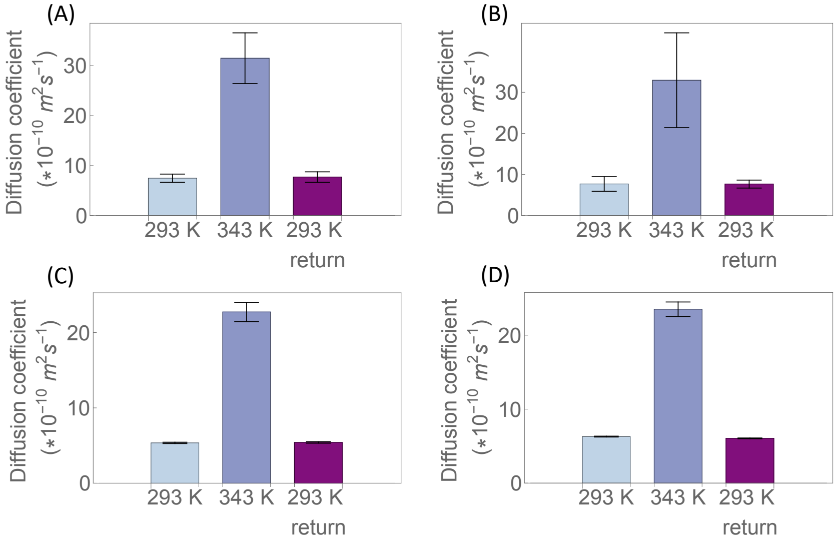

Fig. S20. Diffusion coefficients extracted from ^{31}P DOSY experiments. (A) 100 mM sodium phosphate at pH 4.2, (B) 100 mM potassium phosphate at pH 4.0, (C) 1 M sodium phosphate at pH 4.2, (d) 1 M potassium phosphate at pH 4.1.

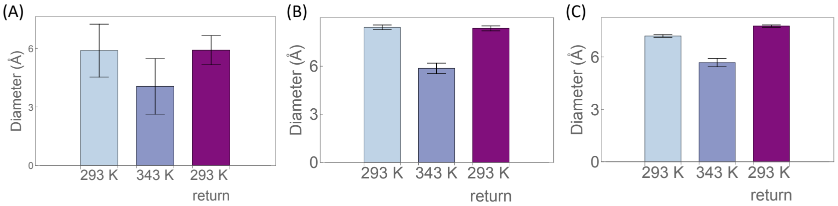

Fig. S21. Hydrodynamic diameters found by the Stokes-Einstein relaxation from diffusion coefficients in S5. (A) 100 mM potassium phosphate, (B) 1 M sodium phosphate, (C) 1 M potassium phosphate.

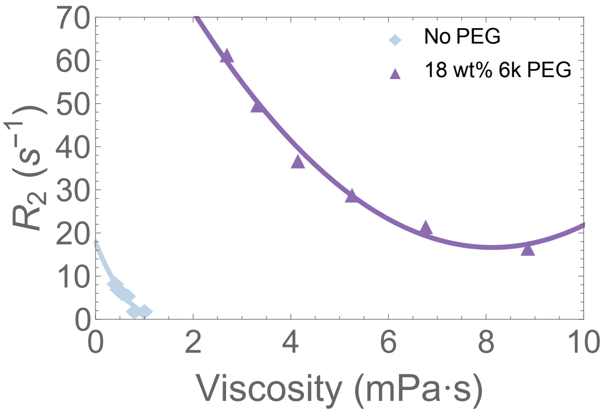

Fig. S22. R_2 relaxation rate as extracted from FWHM for 100 mM potassium phosphate at pH 4.5 in the absence of PEG and for a 18 wt% PEG solution as a function of viscosity (6), showing that viscosity alone cannot explain the effect of PEG on relaxation rates. Solid lines are quadratic fits to data to guide the eye.

References

1. MJ Abraham, et al., GROMACS: High performance molecular simulations through multi-level parallelism from laptops to supercomputers. *SoftwareX* **1-2**, 19–25 (2015).
2. A Bondi, van der waals volumes and radii. *The J. Phys. Chem.* **68**, 441–451 (1964).
3. P Eastman, et al., OpenMM 7: Rapid development of high performance algorithms for molecular dynamics. *PLoS Comput. Biol.* **13**, e1005659 (2017).
4. HC Andersen, Rattle: A "velocity" version of the shake algorithm for molecular dynamics calculations. *J. Comput. Phys.* **52**, 24–34 (1983).
5. S Miyamoto, PA Kollman, Settle: An analytical version of the SHAKE and RATTLE algorithm for rigid water models. *J. Comput. Chem.* **13**, 952–962 (1992).
6. M Rahbari-Sisakht, M Taghizadeh, A Eliassi, Densities and viscosities of binary mixtures of poly(ethylene glycol) and poly(propylene glycol) in water and ethanol in the 293.15-338.15 K temperature range. *J. Chem. & Eng. Data* **48**, 1221–1224 (2003).
